# Altered Cortical Functional Networks in Patients With Schizophrenia and Bipolar Disorder: A Resting-State Electroencephalographic Study

**DOI:** 10.3389/fpsyt.2020.00661

**Published:** 2020-07-17

**Authors:** Sungkean Kim, Yong-Wook Kim, Miseon Shim, Min Jin Jin, Chang-Hwan Im, Seung-Hwan Lee

**Affiliations:** ^1^ J. Crayton Pruitt Family Department of Biomedical Engineering, University of Florida, Gainesville, FL, United States; ^2^ Clinical Emotion and Cognition Research Laboratory, Inje University, Goyang, South Korea; ^3^ Department of Biomedical Engineering, Hanyang University, Seoul, South Korea; ^4^ Institute of Industrial Technology, Korea University, Sejong, South Korea; ^5^ Department of Psychiatry, Wonkwang University Hospital, Iksan, South Korea; ^6^ Department of Psychiatry, Inje University Ilsan Paik Hospital, Ilsan, South Korea

**Keywords:** ipolar disorder, cortical functional network, graph theory, resting-state EEG, schizophrenia

## Abstract

**Background:**

Pathologies of schizophrenia and bipolar disorder have been poorly understood. Brain network analysis could help understand brain mechanisms of schizophrenia and bipolar disorder. This study investigates the source-level brain cortical networks using resting-state electroencephalography (EEG) in patients with schizophrenia and bipolar disorder.

**Methods:**

Resting-state EEG was measured in 38 patients with schizophrenia, 34 patients with bipolar disorder type I, and 30 healthy controls. Graph theory based source-level weighted functional networks were evaluated: strength, clustering coefficient (CC), path length (PL), and efficiency in six frequency bands.

**Results:**

At the global level, patients with schizophrenia or bipolar disorder showed higher strength, CC, and efficiency, and lower PL in the theta band, compared to healthy controls. At the nodal level, patients with schizophrenia or bipolar disorder showed higher CCs, mostly in the frontal lobe for the theta band. Particularly, patients with schizophrenia showed higher nodal CCs in the left inferior frontal cortex and the left ascending ramus of the lateral sulcus compared to patients with bipolar disorder. In addition, the nodal-level theta band CC of the superior frontal gyrus and sulcus (cognition-related region) correlated with positive symptoms and social and occupational functioning scale (SOFAS) scores in the schizophrenia group, while that of the middle frontal gyrus (emotion-related region) correlated with SOFAS scores in the bipolar disorder group.

**Conclusions:**

Altered cortical networks were revealed and these alterations were significantly correlated with core pathological symptoms of schizophrenia and bipolar disorder. These source-level cortical network indices could be promising biomarkers to evaluate patients with schizophrenia and bipolar disorder.

## Introduction

Schizophrenia and bipolar disorder are both major psychiatric disorders. Schizophrenia is frequently characterized by positive and negative symptoms, whereas bipolar disorder is generally characterized by mania and depression (1). Schizophrenia and bipolar disorder have both similarities and differences with respect to neuropsychological and neurophysiological levels. In addition, they have overlapping symptoms, such as psychotic symptoms, disorganized thinking, and depressive symptoms (2–4). However, the pathologies of these two diseases have not yet been revealed (2, 5). Therefore, studies that could help in understanding the pathologies of these two diseases are needed.

Electroencephalography (EEG) can detect the synchronous activity in neuronal populations. Because EEG is mainly produced by post-synaptic potentials, it is susceptible to alterations in neurotransmission secondary to pharmacological manipulations or brain dysfunction (6). Resting state brain activity reflects the baseline status of the brain and has been proposed as a means of exploring the underlying pathophysiological characteristics of psychiatric disorders (7). In addition, unique EEG patterns have been observed in mental disorders during resting state. These patterns were associated with the pathophysiological characteristics of the conditions (8, 9). The brain is active during the resting state and the additional consumption of glucose metabolism with task-related activity is often less than 5%, which is only a small portion of overall brain activity (10). Therefore, resting state analysis is necessary to understand the pathophysiology of mental disorders well.

Previous studies indicate abnormal EEG oscillatory activity during resting state in schizophrenia and bipolar disorder. Schizophrenia has shown increased low frequency power and coherence. Although findings for bipolar disorder have been less well characterized than schizophrenia, bipolar disorder has shown increased low frequency power and decreased alpha frequency power (11–14). In addition, Kam et al. (14) reported that bipolar disorder showed increased high frequency power and coherence while schizophrenia showed increased low frequency connectivity within and across hemispheres.

Recently, an increasing number of researchers have paid attention to changes in the cortical functional network to quantify global and local changes using graph theory (15–17). Graph theory has been introduced recently as a method to construct human brain networks. Brain networks based on graph theoretical approaches could help to understand brain mechanisms of psychiatric disorders including schizophrenia and bipolar disorder. Altered activation of resting-state functional connectivity or networks has been shown in schizophrenia (18–23) and bipolar disorder (24–26). For example, a resting-state fMRI study revealed reduced clustering coefficients (CCs) and reduced probability in high degree hubs for schizophrenia (19). For resting-state EEG studies, Rubinov et al. (18) showed lower CCs and shorter path length (PL) in whole frequency bands in schizophrenia. Furthermore, Kim et al. (26) showed decreased CC and efficiency and increased PL in the alpha band in bipolar disorder.

However, these previous EEG studies mainly conducted sensor-level (electrode-level) connectivity and network analyses. They could therefore not identify specific cortical regions contributing to the alteration of the cortical functional network in schizophrenia or bipolar disorder. In sensor-level analysis, EEG has some limitations such as low spatial resolution due to volume conduction (27), and poor signal-to-noise ratios due to diverse artifacts and noises (28); however, source-imaging can be a good alternative to circumvent these issues. The spatial resolution of EEG, in particular, can be considerably improved by mapping the scalp potential distribution onto the underlying cortical source space *via* source-imaging methods. To the best of our knowledge, no study so far has investigated and compared altered source-level cortical functional networks based on graph theory using resting-state EEG in patients with schizophrenia and bipolar disorder.

In this present study, we investigated cortical functional networks in patients with schizophrenia and bipolar disorder through a source-level weighted network analysis during resting-state EEG. Thus, we could observe the alteration of networks in both specific local cortical regions and the global network pattern. Furthermore, we examined the relationships between cortical network indices and psychiatric, clinical, or cognitive measures, which would help in comprehending the pathologies of schizophrenia and bipolar disorder. We hypothesized that patients with schizophrenia and bipolar disorder would exhibit an altered cortical functional network in both global and nodal levels during resting state, and that the altered cortical network indices such as strength, CC, PL, and efficiency would be significantly associated with psychiatric symptom scales.

## Materials and Methods

### Participants

In total, 102 participants with the ages ranging from 20 to 63 years participated in this study. The participants included patients with schizophrenia [n=38, age: 43.16 ± 11.16 (range: 21–60)] and bipolar disorder [n=34, age: 41.44 ± 12.57 (range: 20–63)] as well as healthy controls [n=30, age: 42.97 ± 12.40 (range: 23–63)]. All patients were evaluated for Axis I (29) and II (30) disorders based on the Structured Clinical Interview for the Diagnostic and Statistical Manual of Mental Disorders, 4th edition (SCID) by a board-certified psychiatrist. No patient had alcohol or drug abuse, mental retardation, a lifetime history of central nervous system, or head injury with loss of consciousness. All patients with bipolar disorder were diagnosed with type I. In addition, 10 patients with bipolar disorder had psychotic symptoms. Most of the patients with schizophrenia were being treated with atypical antipsychotics with or without mood-stabilizing agents (lithium, topiramate, lamotrigine, and sodium valproate) and most patients with bipolar disorder were being treated with atypical antipsychotics and mood-stabilizing agents. Thirty-three patients with schizophrenia were on antipsychotics and six patients with schizophrenia were on mood stabilizers. In terms of patients with bipolar disorder, 30 patients were on antipsychotics and 28 patients were on mood stabilizers. Thirty healthy controls were recruited through the local community *via* flyers and newspapers. An initial screening interview excluded participants with head injury or any personal or family history of psychiatric illness or any identifiable neurological disorder. Following the initial screening, potential healthy controls were interviewed through the SCID for Axis II Psychiatric Disorders (30) and were rejected if they had any psychiatric disorders. All participants signed a written informed consent form approved by the Institutional Review Board of Inje University Ilsan Paik Hospital (2015-07-23).

### Psychiatric, Clinical, and Cognitive Measures

Psychiatric symptoms were evaluated using the Positive and Negative Syndrome Scale (PANSS) for schizophrenia (31) and the Young Mania Rating Scale (YMRS) for bipolar disorder (32). To evaluate neurocognition, the Korean Auditory Verbal Learning Test (K-AVLT) (33) was used. K-AVLT belonging to the Rey-Kim Memory Test (33) is a verbal memory test consisting of five immediate recall trials (trials 1–5), delayed recall trials, and delayed recognition trials. Immediate recall score is the sum of correctly recalled words (trials 1–5). The delayed recall score represents the number of correctly recalled words after a 20 min delay period. The delayed recognition score represents the correctly chosen words from the original list (15 words) which are spoken by the examiner among a list of 50 words after delayed recall. To evaluate functional outcomes, the Social and Occupational Functioning Assessment Scale (SOFAS) (34, 35) was used. The SOFAS was applied as a one-item rating scale from clinician’s judgment of overall level of functioning for Axis V in the Diagnostic and Statistical Manual for Mental Disorders 4th Edition. The SOFAS is a global rating scale, ranging from 0 to 100, for current functioning with lower scores indicating lower functioning (34, 35). In addition, the premorbid IQ was measured using the information test from the Korean Wechsler Adult Intelligence Scale (K-WAIS-IV), age, and education year (36).

### Recording and Preprocessing of Electroencephalography (EEG)

Resting-state EEG data were recorded in a sound-attenuated room, while the participants closed their eyes for 5 min. EEG was recorded with a NeuroScan SynAmps2 amplifier (Compumedics USA, Charlotte, NC, USA) using an extended 10–20 placement scheme *via* 62 Ag-AgCl electrodes mounted on a Quik-Cap. The reference electrode was Cz and the ground electrode was on the forehead. Horizontal electrooculogram (EOG) electrodes were placed at the outer canthus of each eye. Vertical EOG electrodes were located above and below the left eye. The impedance was kept below 5 kΩ. EEG signals were bandpass-filtered from 0.1 to 100 Hz with a 1,000 Hz sampling rate.

Recorded EEG data were preprocessed *via* CURRY 7 (Compumedics USA, Charlotte, NC, USA). EEG data were re-referenced to an average reference. A high pass filter with a cutoff frequency of 1 Hz was applied to the EEG data to remove DC components from the data. Movement artifacts were removed *via* visual inspection of an experienced researcher without prior information regarding the data origin. Eye-movement related artifacts were corrected using a covariance and regression based mathematical procedure implemented in the preprocessing software (37) of CURRY 7. After dividing pre-processed EEG data into 2 s (2,048 points) epochs, all the epochs including significant physiological artifacts (amplitude exceeding ± 75 μV) at any of the 62 electrodes were rejected. In order to exclude any epochs with drowsiness, we calculated the relative power of theta (4–8 Hz) and alpha (8–12 Hz) bands. Then, we rejected any epochs with ratios of the theta band power to the alpha band power exceeding 1, since these epochs were regarded as drowsiness or sleep stage 1 (38–40). Finally, a total of 30 artifact-free epochs were prepared for each participant. The number of 30 epochs was decided by the different number of remaining epochs for each participant after rejecting artifacts and drowsiness, and also because of the previous study reporting acceptable reliability with resting-state EEG data longer than 40 s (41). In addition, basic power spectra were analyzed to compare relative global band powers among the three groups (supplementary materials).

### Source Localization

The depth-weighted minimum L2 norm estimator from the Brainstorm toolbox (http://neuroimage.usc.edu/brainstorm) was used to approximate time series of source activities (42). A three-layer boundary element model from the MNI/Colin 27 anatomy template was used to compute the leadfield matrix. Cortical current density values at 15,002 cortical vertices were evaluated at every time point for each epoch. Noise covariance was calculated by each participant’s whole 30 epochs. Diagonal components in noise covariance were only used to estimate the weight of each individual sensor in the source reconstruction. Following approximating the cortical current density at every time point, 148 nodes were extracted from the Destrieux atlas containing 74 cortical regions in each hemisphere (43). The representative value in each region was assessed by the cortical source of the seed point located in each region based on the Destrieux atlas, which information was provided in the Brainstorm toolbox. The time series of the cortical sources at each of the 148 seed points were bandpass-filtered and divided into six frequency bands including delta (1–4 Hz), theta (4–8 Hz), alpha (8–12 Hz), low beta (12–18 Hz), high beta (18–30 Hz), and gamma (30–55 Hz). The band-pass filtering was applied to each epoch. We employed some techniques to reduce spectral edge artifacts. First, a high pass filter with a cutoff frequency of 1 Hz was applied to the raw EEG data before epoching. Since the filtering process removed DC components of the data, the spectral edge artifacts were mainly mitigated. Second, we used a 3rd-order Butterworth IIR band-pass filter with zero-phase filtering. Low order filter minimized the length where distortion existed and the zero-phase filtering removed half of the distorted signals.

### Connectivity and Network Analysis

Functional connectivity between each pair of nodes was quantified *via* phase-locking values (PLVs) (44). PLVs result in normalized synchronization values ranging from 0 to 1, and thus no further modification is required before applying them to the weighted network analysis. PLV has been known for the fine performance with weighted minimum norm estimation (45) and has been widely used in the network analysis (46–48).

In this study, we performed a graph theory based weighted network analysis (16, 17). The weighted network preserves unique traits of the original network without distortion. A network is composed of several nodes connecting to each other at their edges. In the present study, we selected representative network measures. Four different global-level weighted network indices were assessed. First, “strength” refers to the degree of connection strength in the network. It is estimated by summing the weights of links connected to brain regions. A greater strength value suggests that the whole brain is strongly connected. Second, “CC” indicates the degree to which a node clusters with its neighboring nodes. An increased CC indicates that a network is well segregated between the relevant brain regions. The CC was calculated for the whole network. Third, “PL” indicates the sum of lengths between two nodes within the network, which is related to the speed of information processing. The shortened PL indicates a well-integrated network. Fourth, “efficiency” represents the effectiveness of information processing in the brain; high efficiency indicates rapid information propagation in the network. Weighted nodal CC was also evaluated for each node.

### Statistical Analysis

Chi-squared tests and one-way analysis of variance (ANOVA) were used to investigate differences in demographic characteristics and psychiatric, clinical, and cognitive measures among the three groups. A multivariate ANOVA (MANOVA) was performed to compare the cortical network characteristics at the global level in each frequency band among the three groups, with premorbid IQ as a covariate. Bonferroni corrections with an adjusted *p*-value of 0.05/24 = 0.002083 (four global network measures with six frequency bands) were used to control for multiple comparisons. The same analysis was performed at the nodal level, followed by Bonferroni corrections with an adjusted *p*-value of 0.05/148 = 0.000338 (nodal CCs of 148 nodes). Furthermore, the variables presenting significant differences among the three groups were analyzed using *post hoc* pair-wise comparisons with Bonferroni corrections. Effect sizes were calculated using partial eta squared (η^2^).

A partial Pearson’s correlation was performed between network indices and psychiatric, clinical, or cognitive measures, with a 5,000-bootstrap resampling technique to correct for multiple correlations in each group. The bootstrap test estimated sampling distribution of an estimator by resampling with replacement from the original sample. For the bootstrap validation, the simple random sampling method which was provided in SPSS package was used. The number of the retest was set to 5,000 and the confidence interval was set to 95%. The bootstrap test is a weaker method than the Bonferroni test to solve the multiple-comparison issue. However, the stability and robustness of the bootstrap test have been established by diverse previous studies (49, 50). Further, the bootstrap test has been widely carried out in EEG analysis (51, 52). For the patient groups, the potential effects of medication (equivalent doses of chlorpromazine and sodium valproate) (53) and duration of illness were considered as covariates. The significance level was set at *p* < 0.05 (two-tailed). Statistical analyses were conducted using SPSS 21 (SPSS, Inc., Chicago, IL, USA).

## Results

### Demographic and Psychiatric, Clinical, and Cognitive Characteristics

**Table 1** shows the comparison of demographic and psychiatric, clinical, and cognitive characteristics among the patients with schizophrenia or bipolar disorder and the healthy controls. There were significant differences in premorbid IQ, K-AVLT-trial 5, K-AVLT-delayed recall, and SOFAS. Premorbid IQ showed significant difference among the three groups; healthy controls presented a significantly higher premorbid IQ than patients with schizophrenia or bipolar disorder (100.60 ± 10.64 *vs.* 98.68 ± 8.15 *vs.* 108.23 ± 9.13, *p* < 0.001). The score of the K-AVLT-trial 5 was significantly lower in patients with schizophrenia than in those with bipolar disorder and healthy controls (K-AVLT-trial 5: 8.37 ± 2.79 *vs.* 10.61 ± 2.97 *vs.* 11.57 ± 1.75, *p* < 0.001). The score of the K-AVLT-delayed recall was significantly higher in healthy controls than in patients with schizophrenia or bipolar disorder (K-AVLT-delayed recall: 6.11 ± 3.29 *vs.* 7.97 ± 3.59 *vs.* 9.96 ± 2.01, *p* < 0.001). Furthermore, The SOFAS score was significantly lower in patients with schizophrenia than in those with bipolar disorder (64.54 ± 12.50 *vs.* 72.35 ± 11.76, *p* = 0.009). The order of all scores for group comparison is schizophrenia, bipolar disorder, and healthy controls.

**Table 1:** Demographic characteristics of study participants.

	SZ(N = 38)	BP(N = 34)	HC(N = 30)	*P*	Post-hoc(Bonferroni)
Age (years)	43.16 ± 11.16	41.44 ± 12.57	42.97 ± 12.40	0.810	
Sex				0.141	
Male	16 (42.1)	9 (26.5)	15 (50.0)		
Female	22 (57.9)	25 (73.5)	15 (50.0)		
Premorbid IQ	100.60 ± 10.64	98.68 ± 8.15	108.23 ± 9.13	<0.001	SZ < HC, BP < HC
Education (years)	12.92 ± 2.73	12.79 ± 2.82	14.13 ± 3.51	0.153	
Number of hospitalizations	2.84 ± 3.58	4.12 ± 10.06		0.466	
Duration of illness (years)	12.09 ± 7.55	10.45 ± 6.96		0.371	
Onset age (years)	30.06 ± 10.94	31.26 ± 12.88		0.689	
Dosage of medication* (CPZ equivalent, mg)	319.21 ± 307.81	276.49 ± 410.92			
Dosage of medication* (equivalent to sodium valproate dose, mg)	85.53 ± 207.59	762.82 ± 463.93			
PANSS					
Positive	8.19 ± 4.89	4.56 ± 1.11			
Negative	14.27 ± 5.31	7.97 ± 2.42			
Disorganized	5.68 ± 2.83	3.79 ± 1.04			
Excitation	7.76 ± 3.44	5.03 ± 1.38			
Depression	6.68 ± 1.62	5.71 ± 2.07			
Total	60.92 ± 21.71	41.26 ± 8.42			
YMRS		5.56 ± 6.37			
KAVLT-trial 5	8.37 ± 2.79	10.61 ± 2.97	11.57 ± 1.75	<0.001	SZ < BP, SZ < HC
KAVLT-delayed recall	6.11 ± 3.29	7.97 ± 3.59	9.96 ± 2.01	<0.001	SZ < BP, SZ < HC, BP < HC
SOFAS	64.54 ± 12.50	72.35 ± 11.76		0.009	

SZ, schizophrenia; BP, bipolar disorder; HC, healthy control; CPZ, chlorpromazine; PANSS, positive and negative syndrome scale; YMRS, Young mania rating scale; KAVLT, Korean auditory verbal learning test; HADS, hospital anxiety and depression scale; SOFAS, social and occupational functioning assessment scale.

*The mean CPZ (p = 0.617) and valproate equivalents reflect all patients (p < 0.001), including the patients who were not receiving the medications.

### Global-Level Differences in Cortical Functional Networks

**Table 2** presents the comparison of global-level indices, including strength, CC, PL, and efficiency for each frequency band among the groups with schizophrenia and bipolar disorder and the healthy controls. There were significant differences in the four global-level indices of the theta band. The strength, CC, and efficiency of the theta band were significantly higher in the patients with schizophrenia or bipolar disorder compared to healthy controls (strength: 54.16 ± 2.73 *vs.* 53.31 ± 2.25 *vs.* 51.87 ± 2.07, *p* < 0.001; CC: 0.36 ± 0.02 *vs.* 0.35 ± 0.01 *vs.* 0.34 ± 0.01, *p* = 0.001; efficiency: 0.36 ± 0.02 *vs.* 0.36 ± 0.02 *vs.* 0.35 ± 0.01, *p* < 0.001). On the other hand, the PL of the theta band was significantly lower in patients with schizophrenia or bipolar disorder compared to healthy controls (3.00 ± 0.13 *vs.* 3.03 ± 0.11 *vs.* 3.10 ± 0.11, *p* = 0.001). There was no significant difference between the patient groups for the four network indices of the theta band. Furthermore, there was no significant difference among the three groups in other frequency bands. The order of all network values for group comparison is schizophrenia, bipolar disorder, and healthy controls. The violin plot figures were presented for the distribution of each network measure in the supplementary material (**Supplementary Figure S1**). In addition, the relative global band powers showed a significant difference among the three groups only in the theta band. The relative power of the theta band was significantly higher in the patients with schizophrenia compared to healthy controls (**Supplementary Table S1**).

**Table 2:** Mean and standard deviation values of global network indices including strength, clustering coefficient (CC), path length (PL), and efficiency for each frequency band among the schizophrenia, bipolar disorder, and healthy control groups.

	SZ(N = 38)	BP(N = 34)	HC(N = 30)	Effect size(η^2^)	*F*	P^*^	Post-hoc(Bonferroni)
Delta band							
Strength	62.242 ± 3.427	62.273 ± 3.085	61.498 ± 2.953	0.027	1.303	0.277	
CC	0.414 ± 0.022	0.415 ± 0.021	0.410 ± 0.019	0.024	1.160	0.318	
PL	2.551 ± 0.120	2.540 ± 0.120	2.566 ± 0.118	0.021	0.974	0.381	
Efficiency	0.417 ± 0.023	0.417 ± 0.021	0.412 ± 0.020	0.027	1.303	0.277	
**Theta band**							
**Strength**	54.161 ± 2.727	53.309 ± 2.254	51.865 ± 2.074	0.156	8.594	**<0.001**	SZ > HC, BP > HC
**CC**	0.357 ± 0.017	0.352 ± 0.015	0.343 ± 0.013	0.148	8.095	**0.001**	SZ > HC, BP > HC
**PL**	3.000 ± 0.129	3.032 ± 0.114	3.103 ± 0.110	0.136	7.318	**0.001**	SZ < HC, BP < HC
**Efficiency**	0.362 ± 0.019	0.356 ± 0.015	0.346 ± 0.014	0.156	8.608	**<0.001**	SZ > HC, BP > HC
Alpha band							
Strength	54.634 ± 2.745	53.989 ± 2.591	53.053 ± 2.178	0.064	3.167	0.047	
CC	0.360 ± 0.018	0.356 ± 0.017	0.351 ± 0.014	0.056	2.769	0.068	
PL	2.968 ± 0.130	2.991 ± 0.126	3.033 ± 0.111	0.047	2.288	0.107	
Efficiency	0.365 ± 0.019	0.361 ± 0.018	0.354 ± 0.015	0.064	3.176	0.046	
Low beta band							
Strength	48.215 ± 3.088	48.355 ± 6.175	46.773 ± 3.292	0.047	2.309	0.105	
CC	0.313 ± 0.020	0.315 ± 0.042	0.305 ± 0.021	0.046	2.217	0.115	
PL	3.481 ± 0.194	3.485 ± 0.322	3.571 ± 0.216	0.046	2.231	0.113	
Efficiency	0.322 ± 0.021	0.323 ± 0.042	0.313 ± 0.023	0.047	2.307	0.105	
High beta band							
Strength	41.317 ± 4.594	41.415 ± 4.358	42.645 ± 5.351	0.002	0.092	0.913	
CC	0.261 ± 0.030	0.263 ± 0.030	0.272 ± 0.036	0.004	0.189	0.828	
PL	4.195 ± 0.387	4.180 ± 0.389	4.095 ± 0.489	0.002	0.086	0.918	
Efficiency	0.285 ± 0.031	0.285 ± 0.029	0.292 ± 0.036	0.001	0.043	0.958	
Gamma band							
Strength	30.438 ± 2.152	30.425 ± 1.983	29.938 ± 2.229	0.031	1.509	0.227	
CC	0.183 ± 0.013	0.184 ± 0.012	0.181 ± 0.014	0.025	1.206	0.304	
PL	5.381 ± 0.386	5.391 ± 0.394	5.512 ± 0.427	0.042	2.038	0.136	
Efficiency	0.230 ± 0.017	0.229 ± 0.016	0.224 ± 0.017	0.048	2.336	0.102	

*The p-value was adjusted via Bonferroni correction with 0.05/24 = 0.002083.

SZ, schizophrenia; BP, bipolar disorder; HC, healthy control. Values are in bold to visually highlight them in the table.

### Nodal-Level Differences in Cortical Functional Networks

Based on the significant difference in the global theta-band CCs among the three groups, we determined to investigate possible differences at the local level in the theta band. The nodal CCs among the three groups were significantly different in 23 regions. Firstly, the nodal CCs of the schizophrenia and bipolar disorder groups were significantly higher compared to the healthy controls in eight regions (left anterior part of the cingulate gyrus and sulcus: 0.37 ± 0.02 *vs.* 0.36 ± 0.02 *vs.* 0.35 ± 0.02, *p* < 0.001; right anterior part of the cingulate gyrus and sulcus: 0.37 ± 0.02 *vs.* 0.36 ± 0.02 *vs.* 0.35 ± 0.02, *p* < 0.001; right frontomarginal gyrus and sulcus: 0.35 ± 0.02 *vs.* 0.34 ± 0.02 *vs.* 0.33 ± 0.01, *p* < 0.001; right middle frontal gyrus: 0.34 ± 0.02 *vs.* 0.33 ± 0.01 *vs.* 0.32 ± 0.01, *p* < 0.001; left superior frontal gyrus: 0.33 ± 0.02 *vs.* 0.33 ± 0.01 *vs.* 0.32 ± 0.01, *p* < 0.001; right superior frontal gyrus: 0.34 ± 0.02 *vs.* 0.34 ± 0.01 *vs.* 0.33 ± 0.01, *p* < 0.001; right middle occipital gyrus: 0.34 ± 0.01 *vs.* 0.34 ± 0.02 *vs.* 0.33 ± 0.01, *p* < 0.001; right superior frontal sulcus: 0.34 ± 0.02 *vs.* 0.33 ± 0.01 *vs.* 0.32 ± 0.01, *p* < 0.001).

Secondly, the nodal CCs of the schizophrenia group were significantly higher than those of the bipolar disorder group and the healthy controls in five regions (left opercular part of the inferior frontal gyrus: 0.35 ± 0.02 *vs.* 0.34 ± 0.01 *vs.* 0.33 ± 0.01, *p* < 0.001; left orbital part of the inferior frontal gyrus: 0.35 ± 0.02 *vs.* 0.34 ± 0.01 *vs.* 0.33 ± 0.01, *p* < 0.001; left triangular part of the inferior frontal gyrus: 0.35 ± 0.02 *vs.* 0.34 ± 0.01 *vs.* 0.33 ± 0.01, *p* < 0.001; left vertical ramus of the anterior segment of the lateral sulcus: 0.36 ± 0.02 *vs.* 0.34 ± 0.02 *vs.* 0.34 ± 0.01, *p* < 0.001; left inferior frontal sulcus: 0.35 ± 0.02 *vs.* 0.34 ± 0.02 *vs.* 0.33 ± 0.01, *p* < 0.001).

**Figure 1:**
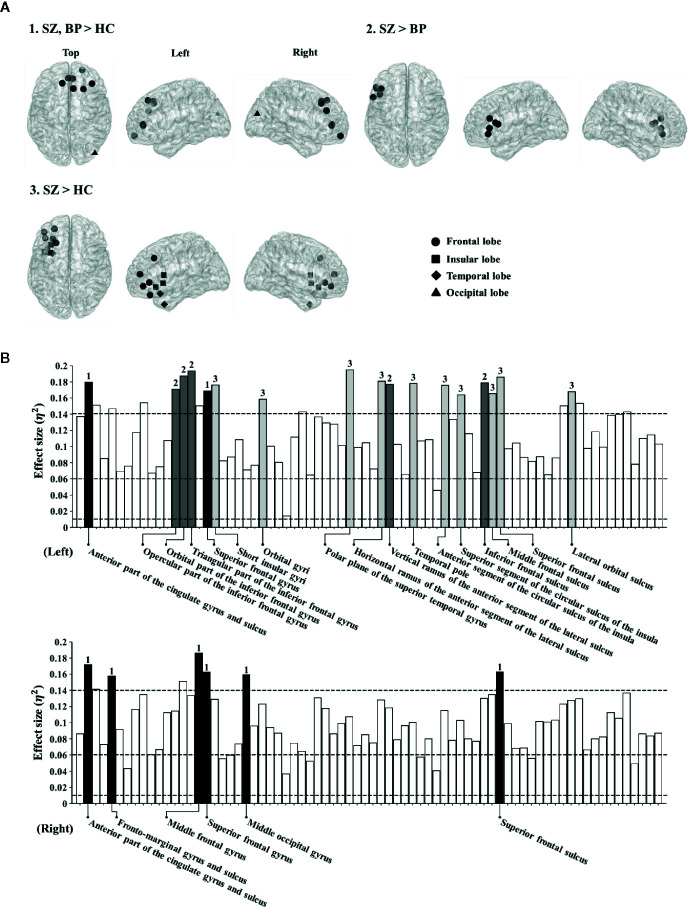
**(A)** Brain regions showing significantly different nodal clustering coefficients (CCs) of the theta frequency band among patient groups and healthy controls. **(B)** Effect sizes of differences in nodal CCs of the theta frequency band among patient groups and healthy controls. Each bar indicates the effect size at each node. The upper bars indicate 74 regions in the left hemisphere and the lower bars indicate 74 regions in the right hemisphere. For reference, three dotted lines are drawn for small (0.01), medium (0.06), and large (0.14) effect sizes. The bars with numbers reveal significant differences among patient groups and the healthy controls (The *p*-value was adjusted *via* Bonferroni correction with 0.05/148 = 0.000338). The number “1” denotes brain regions where patient groups show significant differences from healthy controls. The number “2” denotes brain regions where patients with schizophrenia show significant differences from those with bipolar disorder. The number “3” denotes brain regions where patients with schizophrenia show significant differences from healthy controls.

Thirdly, the nodal CCs of the schizophrenia group were significantly higher compared to the healthy controls in 10 regions (left short insular gyri: 0.37 ± 0.02 *vs.* 0.36 ± 0.02 *vs.* 0.35 ± 0.01, *p* < 0.001; left orbital gyri: 0.38 ± 0.02 *vs.* 0.37 ± 0.02 *vs.* 0.36 ± 0.02, *p* < 0.001; left polar plane of the superior temporal gyrus: 0.38 ± 0.02 *vs.* 0.36 ± 0.02 *vs.* 0.35 ± 0.02, *p* < 0.001; left horizontal ramus of the anterior segment of the lateral sulcus: 0.36 ± 0.02 *vs.* 0.35 ± 0.02 *vs.* 0.34 ± 0.01, *p* < 0.001; left temporal pole: 0.38 ± 0.02 *vs.* 0.37 ± 0.02 *vs.* 0.36 ± 0.02, *p* < 0.001; left anterior segment of the circular sulcus of the insula: 0.38 ± 0.02 *vs.* 0.37 ± 0.02 *vs.* 0.36 ± 0.02, *p* < 0.001; left superior segment of the circular sulcus of the insula: 0.38 ± 0.03 *vs.* 0.37 ± 0.02 *vs.* 0.36 ± 0.02, *p* < 0.001; left middle frontal sulcus: 0.35 ± 0.03 *vs.* 0.34 ± 0.02 *vs.* 0.33 ± 0.01, *p* < 0.001; left superior frontal sulcus: 0.35 ± 0.02 *vs.* 0.34 ± 0.01 *vs.* 0.33 ± 0.02, *p* < 0.001; left lateral orbital sulcus: 0.36 ± 0.02 *vs.* 0.35 ± 0.02 *vs.* 0.34 ± 0.01, *p* < 0.001) (**Figure 1**). The order of all nodal CC values for group comparison is schizophrenia, bipolar disorder, and healthy controls.

### Correlation Between Network Indices and Psychiatric, Clinical, or Cognitive Characteristics

**Figure 2:**
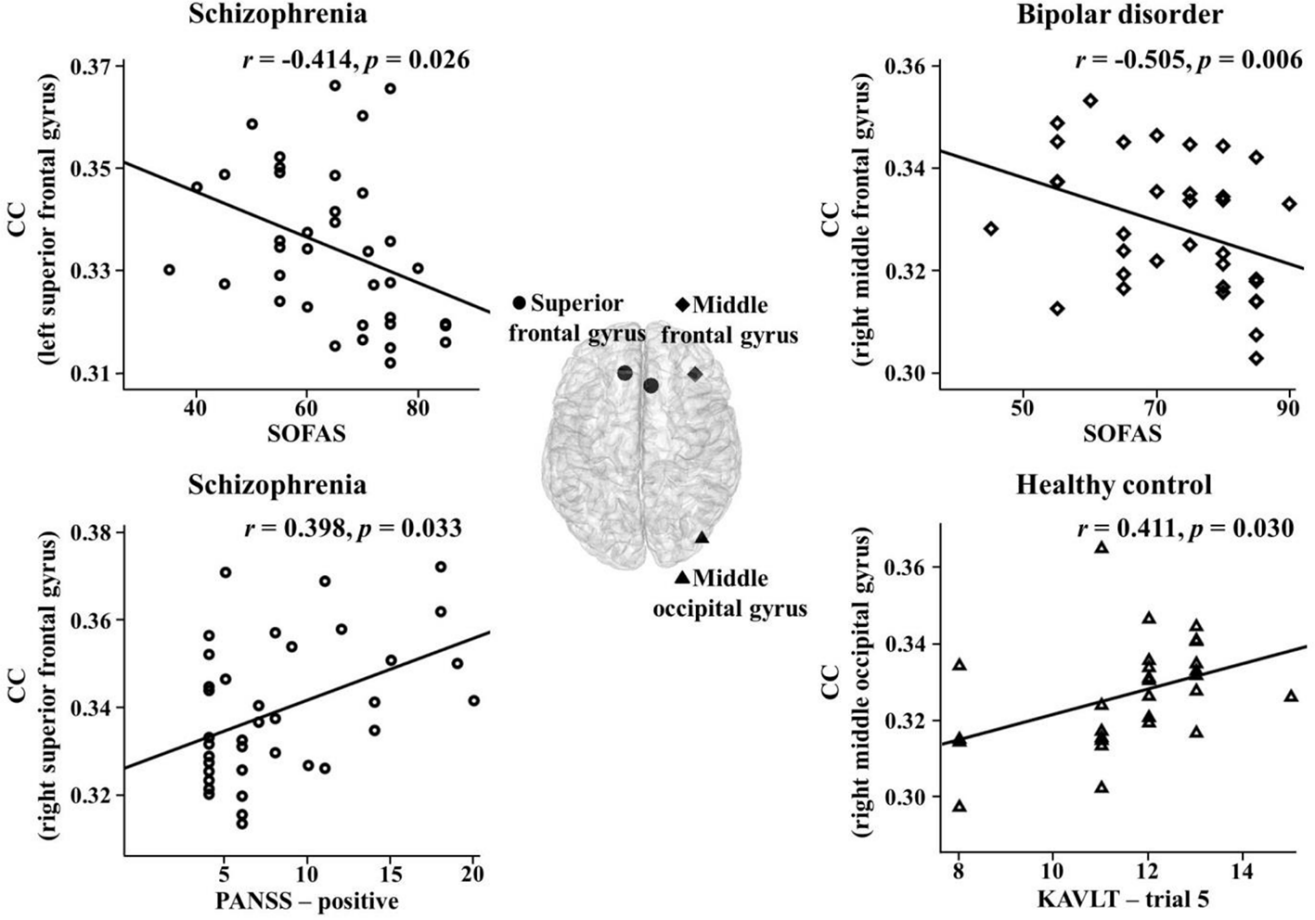
Correlations between nodal clustering coefficients (CCs) and psychiatric, clinical, or cognitive measures in the theta band for each group. SOFAS, social and occupational functioning assessment scale; PANSS, positive and negative syndrome scale; KAVLT, Korean auditory verbal learning test.

The correlations between the network indices of the global and nodal levels and psychiatric, clinical, or cognitive measures were investigated in the theta band. There were significant correlations between them in the three groups. In the schizophrenia group, the nodal CCs in the right superior frontal gyrus and sulcus significantly positively correlated with positive PANSS symptoms (r = 0.398, *p* = 0.033; r = 0.397, *p* = 0.033). In addition, there was a significant negative correlation between the nodal CC in the left superior frontal gyrus and SOFAS scores (r = -0.414, *p* = 0.026). The PL significantly positively correlated with the K-AVLT-delayed recall (r = 0.390, *p* = 0.033). Furthermore, the nodal CC in the right superior frontal sulcus significantly negatively correlated with the K-AVLT-delayed recall (r = -0.368, *p* = 0.045). In the bipolar disorder group, there was a significant negative correlation between the nodal CC in the right middle frontal gyrus and SOFAS scores (r = -0.505, *p* = 0.006). In the healthy controls, the nodal CCs in the left triangular part of the inferior frontal gyrus and the right middle occipital gyrus significantly positively correlated with the K-AVLT-trial 5 (r = 0.391, *p* = 0.040; r = 0.411, *p* = 0.030) (**Figure 2**).

Furthermore, the relationships between the medication dosage of antipsychotics and mood stabilizers and EEG network indices or clinical-neurocognitive measures were investigated. In patients with schizophrenia, there were significant positive correlations between the dose of mood stabilizers and nodal CCs in left orbital gyri, left polar plane of the superior temporal gyrus, and left anterior segment of the circular sulcus of the insula (r = 0.327, *p* = 0.045; r = 0.328, *p* = 0.045; r = 0.321, *p* = 0.049). There were no significant correlations between the dose of antipsychotics and EEG parameters or symptoms. In patients with bipolar disorder, there were no significant correlations between medication doses and EEG parameters or symptoms.

## Discussion

This study evaluated cortical functional networks during resting-state EEG in patients with schizophrenia or bipolar disorder compared to healthy controls. We only observed significant differences between these groups in the theta band. First, at the global level, strength, CC, and efficiency were significantly higher, while PL was lower, in both patient groups compared to healthy controls. Second, at the nodal level, the CCs, mostly in the frontal lobe, were significantly higher in both patient groups; in particular, patients with schizophrenia showed higher nodal CCs in the left inferior frontal cortex and the left ascending ramus of the lateral sulcus, compared to patients with bipolar disorder. Third, the nodal-level theta-band CC of the superior frontal gyrus and sulcus (cognition-related region) correlated with positive PANSS symptoms, SOFAS scores, and verbal memory in patients with schizophrenia, while that of the middle frontal gyrus (emotion-related region) correlated with SOFAS scores in patients with bipolar disorder.

Schizophrenia and bipolar disorder have been revealed to have abnormalities in the structural and functional connectivity at the network level. Previous EEG studies have reported altered resting-state networks in patients with schizophrenia and bipolar disorder. Rubinov et al. (18) showed lower CC and shorter PL in whole frequency bands in schizophrenia. Jalili and Knyazeva et al. (54) found broad decreased synchronizability in several frequency bands including theta, alpha, beta, and gamma bands. In addition, Kim et al. (26) showed decreased CC and efficiency whereas increased PL in the alpha band in bipolar disorder. Resting-state fMRI studies to examine brain network topology in schizophrenia showed global and nodal topological changes with decreased CC and increased efficiency (55) and reduced CC and reduced probability in high degree hubs (19). Furthermore, a resting-state fMRI study with bipolar disorder reported regional abnormalities in default mode and sensorimotor networks (56). In terms of structural networks, structural network studies using diffusion tensor imaging have reported increased PL or decreased efficiency in patients with schizophrenia (20, 57). In bipolar disorder, structural brain networks from diffusion tensor imaging exhibited lower CCs and efficiency and longer PL (58, 59). These previous findings support our results that the patients with schizophrenia and bipolar disorder showed abnormal topological organization of cortical functional networks.

Although previous EEG studies did not show a consensus of a specific frequency band abnormality, our study revealed the theta band abnormality in schizophrenia and bipolar disorder patients. Theta oscillations index learning, memory, and cognitive performance (60, 61). Altered theta-band activities have repeatedly been reported in patients with schizophrenia and bipolar disorder. Resting-state EEG studies consistently reported that patients with schizophrenia show augmented theta-band power (9, 11). A study investigating source functional connectivity during resting-state EEG reported that schizophrenia patients had greater functional connectivity than healthy controls in theta band. Particularly, the patients with longer duration of illness showed higher theta band connectivity in frontal regions compared to those with shorter duration (62). Also, according to the review of the bipolar disorder literature, increased theta power of resting-state EEG is one of the most robust findings in bipolar disorder (13). Thus, abnormal theta oscillation might have a key role in schizophrenia and bipolar disorder. Moreover, the higher strength, CC, and efficiency as well as lower PL of the theta band during resting state in this study seem to represent the poor functioning of the network, which might be associated with potentially excessive or inefficient neural processing in patients with schizophrenia and bipolar disorder (14, 63, 64).

The nodal CCs of the schizophrenia and bipolar disorder group were significantly higher in the anterior cingulate cortex, which connects limbic structures with the prefrontal cortex. The anterior cingulate cortex plays an important role in frontolimbic networks regulating cognitive and emotional functions (65) in patients with schizophrenia (66) and bipolar disorder (67). The volume reduction and cortical thinning of this region have been consistently discovered in schizophrenia (68, 69) and bipolar disorder (67, 70). Structural brain abnormalities in the frontal lobe appear commonly in schizophrenia and bipolar disorder, indicating a biological feature shared by both patient groups (69, 71).

Interestingly, the nodal CCs were significantly higher in left inferior frontal cortex and the left ascending ramus of the lateral sulcus in patients with schizophrenia compared to those with bipolar disorder and healthy controls. The left inferior frontal gyrus plays a significant role in executive functions, such as cognitive inhibition (72) and semantic and language function (73). Volumetric reduction and cortical thinning of this region have been reported in schizophrenia (73, 74). The lateral sulcus is involved in language function (75). Patients with schizophrenia showed reduced lateral sulcus length asymmetry, indicating that schizophrenia is a neurodevelopmental disorder causing impaired cerebral lateralization (76–78). According to the preexisting notion, our findings might imply that executive and language functions are vulnerable in patients with schizophrenia compared to those with bipolar disorder and healthy controls.

Additionally, the nodal CCs of the schizophrenia group were significantly higher in the left insular cortex and the left middle frontal sulcus, compared to healthy controls. The insular cortex belongs to the limbic region that plays an important role in integrating perceptual experiences and affect to generate balanced behavior (79). There is robust evidence of functional and structural abnormalities of this region in schizophrenia (80–83). In addition, abnormalities in cortical gyrification of the left middle frontal sulcus have been reported in chronic patients with schizophrenia with auditory hallucinations (84).

In the schizophrenia group, the nodal CCs of the right superior frontal gyrus and sulcus were positively correlated with positive PANSS symptoms. The nodal CC of the left superior frontal gyrus was negatively correlated with social and occupational functioning. The PL was positively correlated with delayed verbal memory. In addition, the nodal CC in the right superior frontal sulcus was negatively correlated with delayed verbal memory. The superior frontal gyrus has been known to be involved in various processes relying on cognitive control, such as set-switching (85), working memory (86), and complex problem solving (87). A greater deactivation in the bilateral superior frontal gyrus has been associated with positive symptom severity in patients with schizophrenia (88). In addition, other studies that investigated the correlation between the superior frontal region and PANSS score in patients with schizophrenia showed significant correlations between only the PANSS positive score and the superior frontal region of gray matter volume or fractional anisotropy (89–91). These findings support our results indicating that the right superior frontal region is more related with PANSS positive score in patients with schizophrenia. The cortical thickness of the superior frontal gyrus is significantly decreased in patients with schizophrenia compared to healthy controls (92, 93), and decreased cortical thickness in this region has been shown to be related to functioning impairments (93). Furthermore, it is well known that verbal memory is the most impaired field of cognitive function in schizophrenia (94, 95).

In patients with bipolar disorder, the nodal CC of the right middle frontal gyrus was negatively correlated with social and occupational functioning. The middle frontal cortex has been suggested to be particularly implicated in the down-regulation of the emotional response (96, 97). The dorsolateral prefrontal cortex, which lies in the middle frontal gyrus, has been shown to be associated with the motivation factor and interest in social functioning (98). Furthermore, this region is one component of the neural network playing a key role in psychosocial functioning in bipolar disorder (99). Although the correlation between nodal CC of the right middle frontal gyrus and social and occupational functioning was observed in patients with bipolar disorder, the correlation between network measures of the right middle frontal gyrus and YMRS was not shown. This could be explained by the following reasons. Social functioning including social cognition demands cognitive and emotional capacity (100). Previous studies have shown that activities involving the right middle frontal cortex were correlated with emotion (101, 102). In addition, the relationship between YMRS and emotion has been only observed in manic patients, but not in euthymic and depressed phase patients (101, 103, 104). Since the bipolar disorder patients which participated in this study were almost all in euthymic phase, it was possible that the YMRS score was not correlated with emotion. These issues could be the reasons why patients showed only correlations with social and occupational functioning.

In healthy controls, the nodal CCs in the left inferior frontal gyrus and right middle occipital gyrus were positively correlated with immediate verbal memory. Previous studies indicate that increased theta-band oscillations of healthy controls are a remarkable EEG indicator of good cognitive functions such as attention and memory (105). In addition, high theta power has been associated with better cognitive functioning, including immediate and delayed verbal recall in healthy adults (106). Notably, the inferior frontal gyrus is associated with speech production and verbal working memory (107). One study reported that the activation in the middle occipital gyrus was associated with auditory verbal memory (108).

Taken together, our study reveals that the nodal CCs of the superior frontal gyrus and sulcus (relatively cognition-related region) correlate with positive PANSS symptoms, SOFAS scores, and verbal memory in patients with schizophrenia, while those of the middle frontal gyrus (relatively emotion-related region) correlate with SOFAS scores in patients with bipolar disorder. The impairment of brain function in these regions would affect impaired cortical functional networks. In addition, brain dysfunction could lead to abnormal changes in clinical and cognitive measures. The nodal CCs might be predictable biomarkers of psychiatric symptoms.

This study has the following limitations. First, most of the patients were chronic and were taking atypical antipsychotics and mood-stabilizing agents. Second, we did not use individual head models for EEG source imaging and source analysis of scalp-derived EEG might be inherently limited in its precision of spatial localization. Third, the PLVs did not exclude zero-degree phase lags, which could be caused by volume conduction. Even though we estimated connectivity from source time series, volume conduction might still be present. Despite these limitations, this study was the first attempt to compare the source-level cortical functional networks in schizophrenia and bipolar disorder using resting-state EEG. Our results demonstrate altered cortical networks at both global and nodal levels of the theta band in patients with schizophrenia or bipolar disorder. In addition, we found significant correlations between cortical network states and symptom severity scores. These source-level cortical network indices could be promising biomarkers to evaluate patients with schizophrenia and bipolar disorder.

## Data Availability Statement

The datasets generated for this study are available on request to the corresponding author.

## Ethics Statement

The studies involving human participants were reviewed and approved by the Institutional Review Board of Inje University Ilsan Paik Hospital (2015-07-23). The patients/participants provided their written informed consent to participate in this study.

## Author Contributions

SK analyzed the data and wrote the paper. Y-WK collected the data and wrote the paper. MS and MJJ collected and analyzed the data. S-HL designed the study and wrote the paper. S-HL and C-HI reviewed and revised the paper. All authors contributed to the article and approved the submitted version.

## Funding

This work was supported by a grant from the Korea Science and Engineering Foundation (KOSEF), funded by the Korean government (NRF-2018R1A2A2A05018505).

## Conflict of Interest

The authors declare that the research was conducted in the absence of any commercial or financial relationships that could be construed as a potential conflict of interest.
