# Supplementary material for: Altered Cortical Functional Networks in Patients With Schizophrenia and Bipolar Disorder: A Resting-State Electroencephalographic Study
Source: Front Psychiatry. 2020 Jul 17;11:661. doi: 10.3389/fpsyt.2020.00661 (PMC7388793; doi:10.3389/fpsyt.2020.00661)
Supplement: Supplementary file 1 [file DataSheet_1.pdf]

Supplementary Figure S1. Violin plot figures of the global level network indices

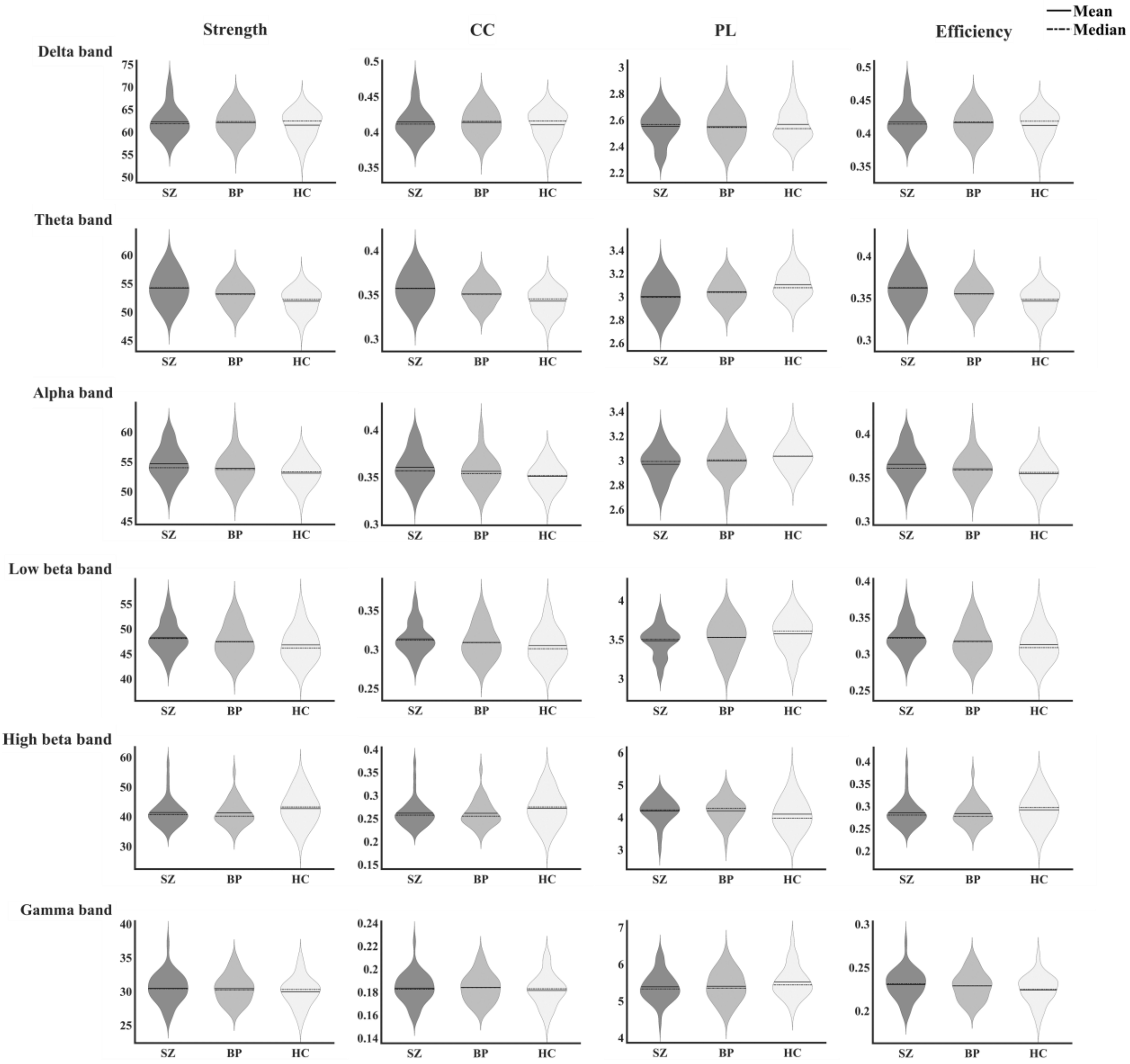

Materials and Methods

Quantitative EEG analysis

The recorded EEG data were preprocessed using CURRY 7 (Compumedics USA, Charlotte, NC, USA). The EEG data were re-referenced to an average reference. Gross artifacts such as movement artifacts were rejected by visual inspection by an experienced researcher with no prior information regarding the data origin. Eye-movement related artifacts were corrected using the mathematical procedure implemented in the preprocessing software (Semlitsch et al., 1986) of CURRY 7. After dividing pre-processed EEG data into epochs with a length of approximately 2 seconds (2,048 points), any epochs including significant physiological artifacts (amplitude exceeding ± 75 μV) at any site among the 62 electrodes were rejected. In order to exclude any epochs with drowsiness, we calculated the relative power of theta (4-8 Hz) and alpha (8-12 Hz) bands. Then, we rejected any epochs with ratios of the theta band power to the alpha band power exceeding 1, since these epochs were regarded as drowsiness or sleep stage 1 (Strijkstra et al., 2003;Eoh et al., 2005;Jap et al., 2009). Finally, a total of 30 epochs, each with a length of approximately 2 seconds (2,048 points), were prepared for each participant. Fast Fourier transformation was performed on 62 electrodes divided into six frequency bands: delta (1-4 Hz), theta (4-8 Hz), alpha (8–12 Hz), low beta (12–18 Hz), high beta (18–30 Hz), and gamma (30–55 Hz). The relative power of each electrode was calculated by dividing each band power by the total power of the electrode. Then, relative global band powers were averaged over 62 electrodes (Gianotti et al., 2007).

Statistical analysis

A multivariate ANOVA (MANOVA) was conducted to compare the relative global band powers for each frequency band among the three groups, with premorbid IQ as a covariate. The variables showing significant differences among the three groups were further analyzed using

post hoc pair-wise comparisons, including Bonferroni corrections. Effect sizes were expressed as partial eta squared (η^2).

Results

Quantitative EEG band power

Supplementary Table S1 shows the comparison of relative global band powers of each frequency band among the groups with schizophrenia and bipolar disorder and the healthy controls. There was a significant difference only in the theta band. The relative power of the theta band was significantly higher in the patients with schizophrenia compared to healthy controls. There was no significant difference between the patient groups or between the patients with bipolar disorder and healthy controls for the relative global power of the theta band.

Supplementary Table S1. Mean and standard deviation values of relative global band powers in each frequency band among the schizophrenia, bipolar disorder, and healthy control groups.

	SZ (N = 38)	BP (N = 34)	HC (N = 30)	Effect size (η^2)	*F*	*P*	Post-hoc (Bonferroni)
Relative power							
Delta band	18.02 ± 10.46	14.76 ± 6.92	14.11 ± 5.58	0.032	1.542	0.219	
Theta band	15.70 ± 5.52	13.52 ± 5.24	11.82 ± 3.80	0.074	3.722	**0.028**	SZ > HC
Alpha band	43.57 ± 9.30	43.92 ± 12.22	50.60 ± 13.61	0.035	1.666	0.195	
Low beta band	9.34 ± 3.57	11.68 ± 5.77	9.68 ± 4.04	0.048	2.331	0.103	
High beta band	9.31 ± 4.51	11.72 ± 5.76	9.85 ± 5.32	0.030	1.428	0.245	
Gamma band	4.07 ± 3.55	4.39 ± 2.60	3.95 ± 2.44	0.001	0.053	0.948	

SZ: schizophrenia; BP: bipolar disorder; HC: healthy control.

References

Eoh, H.J., Chung, M.K., and Kim, S.-H. (2005). Electroencephalographic study of drowsiness in simulated driving with sleep deprivation. *International Journal of Industrial Ergonomics* 35**,** 307-320.

Gianotti, L.R., Künig, G., Lehmann, D., Faber, P.L., Pascual-Marqui, R.D., Kochi, K., and Schreiter-Gasser, U. (2007). Correlation between disease severity and brain electric LORETA tomography in Alzheimer's disease. *Clinical neurophysiology* 118**,** 186-196.

Jap, B.T., Lal, S., Fischer, P., and Bekiaris, E. (2009). Using EEG spectral components to assess algorithms for detecting fatigue. *Expert Systems with Applications* 36**,** 2352-2359.

Semlitsch, H.V., Anderer, P., Schuster, P., and Presslich, O. (1986). A solution for reliable and valid reduction of ocular artifacts, applied to the P300 ERP. *Psychophysiology* 23**,** 695-703.

Strijkstra, A.M., Beersma, D.G., Drayer, B., Halbesma, N., and Daan, S. (2003). Subjective sleepiness correlates negatively with global alpha (8–12 Hz) and positively with central frontal theta (4–8 Hz) frequencies in the human resting awake electroencephalogram. *Neuroscience letters* 340**,** 17-20.
